# Effects of Elevated Tropospheric Ozone Concentration on the Bacterial Community in the Phyllosphere and Rhizoplane of Rice

**DOI:** 10.1371/journal.pone.0163178

**Published:** 2016-09-19

**Authors:** Yoshiaki Ueda, Katharina Frindte, Claudia Knief, Md Ashrafuzzaman, Michael Frei

**Affiliations:** 1 Institute of Crop Science and Resource Conservation (INRES) – Plant Nutrition, University of Bonn, Bonn, Germany; 2 Institute of Crop Science and Resource Conservation (INRES) – Molecular Biology of the Rhizosphere, University of Bonn, Bonn, Germany; Nederlands Instituut voor Ecologie, NETHERLANDS

## Abstract

Microbes constitute a vital part of the plant holobiont. They establish plant-microbe or microbe-microbe associations, forming a unique microbiota with each plant species and under different environmental conditions. These microbial communities have to adapt to diverse environmental conditions, such as geographical location, climate conditions and soil types, and are subjected to changes in their surrounding environment. Elevated ozone concentration is one of the most important aspects of global change, but its effect on microbial communities living on plant surfaces has barely been investigated. In the current study, we aimed at elucidating the potential effect of elevated ozone concentrations on the phyllosphere (aerial part of the plant) and rhizoplane (surface of the root) microbiota by adopting next-generation 16S rRNA amplicon sequencing. A standard *japonica* rice cultivar Nipponbare and an ozone-tolerant breeding line L81 (Nipponbare background) were pre-grown in a greenhouse for 10 weeks and then exposed to ozone at 85 ppb for 7 h daily for 30 days in open top chambers. Microbial cells were collected from the phyllosphere and rhizoplane separately. The treatment or different genotypes did not affect various diversity indices. On the other hand, the relative abundance of some bacterial taxa were significantly affected in the rhizoplane community of ozone-treated plants. A significant effect of ozone was detected by homogeneity of molecular variance analysis in the phyllosphere, meaning that the community from ozone-treated phyllosphere samples was more variable than those from control plants. In addition, a weak treatment effect was observed by clustering samples based on the Yue and Clayton and weighted UniFrac distance matrices among samples. We therefore conclude that the elevated ozone concentrations affected the bacterial community structure of the phyllosphere and the rhizosplane as a whole, even though this effect was rather weak and did not lead to changes of the function of the communities.

## Introduction

Plants are colonized by a large number of microorganisms in nature. Among the plant-colonizing microbes, bacteria represent by far the most dominant domain [1–3], with numbers ranging from 10^6^ to 10^7^ bacteria /cm^2^ in the phyllosphere (leaf surface) and from 10^6^ to 10^9^ bacteria /g soil in the rhizosphere [1,4]. These microorganisms utilize metabolites from plants, establish plant-microbe interactions and affect the surrounding environment as well as the growth and fitness of host plants. Their interactions with the host plants can be beneficial, neutral or harmful [5]. For instance, some microbes such as symbiotic rhizobial bacteria enhance plant growth under nutrient deficiency [1,6]. In contrast, negative plant-microbe interactions involve pathogens, which cause detrimental effects on the host plants [1,5,7]. The plant-colonizing microbes obtain nutrients from the host plant and form their habitat on the plant surface (epiphytes) and/or inside the plants (endophytes). Microbial communities are highly influenced by diverse environmental parameters, including nutrient availability, geographical location and soil type, climatic conditions and further habitat-specific factors such as radiation in the phyllosphere [1,4]. Thus, the microbial community composition is variable as plants grown under various environmental conditions recruit different sets of microbes leading to variable feedback on plant performance and plant-microbe interactions [1,4].

The environments that plants grow in are changing at an unprecedented rate since the last century due to intensive agricultural land use and global climate change [8–10]. These shifts in the environment can affect the microbial communities inhabiting the plant surfaces, and may further lead to secondary effects on the surrounding environment and the host plant. For instance, long-term application of inorganic fertilizer induced changes in the rhizosphere microbial community, especially the relative abundance of some taxa and the diversity of the soil bacteria [11]. Similarly, the rhizosphere microbial community and carbon allocation was altered under elevated atmospheric CO_2_ concentrations, which implicates changes of carbon flow paths in the rhizosphere and a larger effect on the terrestrial ecosystem in the future [12]. These reports highlight the effect of environmental conditions on the plant-associated microbial community and its function, as well as potential secondary effects.

One important aspect of global change is elevated concentrations of tropospheric ozone [9]. This air pollutant is formed from precursor gases such as nitric oxide, carbon monoxide and volatile organic compounds, and its concentration has been rising steadily along with the increasing emission of these precursor gases [13,14]. The background concentration of tropospheric ozone was around 20 ppb in the beginning of the 20^th^ century [15], but the current background concentration has reached 60 ppb in many parts of the world, periodically reaching 80 ppb as a monthly average in some parts of Asia [15,16]. These concentrations are far beyond the critical level for plants [17] and damage cultivated crops and natural vegetation [18,19]. This trend will exacerbate in the future, especially in Asian countries, due to continuing industrialization and rising precursor gas emissions [20].

The effects of elevated tropospheric ozone on plant-based ecosystems and individual plants are fairly well understood due to intensive research during the past decades [21–24]. In contrast, we have only very limited knowledge of the effect of ozone on plant-associated microbes. A few previous studies revealed that ozone decreases the phylogenetic diversity of bacterial and archaeal communities in the rhizosphere under elevated ozone concentration in rice fields [25–27]. However, these analyses were limited to the rhizosphere soil, and little attention has been paid to the microbes directly colonizing the plant surface (*i*.*e*. phyllosphere and rhizoplane). Especially the microorganisms in the phyllosphere may be more strongly affected by elevated ozone concentrations, as they are in direct contact with higher ozone concentrations, while ozone concentrations in soil are very low [28]. Even though earlier reports suggested that ozone negatively affects bacteria at high concentration when they exist alone in the air [29,30], its effect at moderate concentration on plant epiphytes is largely unknown. Considering the above-mentioned vital roles of microbes and possible effects on the ecosystem, more investigation is required to understand this poorly explored aspect of global change and its possible implication for agricultural production.

In the current study, we aimed at assessing the effect of ozone on the microbial community living in association with rice plants, which is presumably the most widely grown crop in the areas highly polluted by tropospheric ozone. We focussed our analysis on the domain of bacteria, which is the most dominant group of microorganisms in the phyllosphere [1,2,4] and rhizoplane/rhizosphere [3,31,32]. We analysed the community of the phyllosphere-associated bacteria and rhizoplane-associated bacteria using the high-throughput 16S rRNA amplicon sequencing strategy [33], which enables culture-independent and deep analysis of the target microbial community. To examine if ozone sensitivity of the host plant influences the effect on the microbial community, we used contrasting genotypes, *i*.*e*. a standard *japonica* type rice cultivar Nipponbare (NB) and an ozone-tolerant breeding line L81 [34], which carries two ozone-tolerant chromosomal introgressions from an *aus* landrace Kasalath [35,36] in the genetic background of NB. We treated these plants with a realistic concentration of ozone for 30 d and analysed the bacterial communities in the phyllosphere and rhizoplane. For each bacterial community, we evaluated; 1) species diversity, 2) community structure and relative abundance of each species, and 3) molecular and functional properties.

## Materials and Methods

### Plant materials and growth condition

Two rice genotypes were used for the study and grown in a greenhouse from March to June 2015. The seeds of NB and L81 were sterilized with 5% NaClO for 5 min, rinsed with deionized water 5 times and imbibed at 28°C in the dark on a petri dish with wet paper. After 3 d, the seedlings were transferred to a greenhouse (located in Bonn, Germany; 50.7°N, 7.1°E) under natural light and grown for another 11 d on a floating netted styrofoam tray in distilled water. Additional supplementary light was employed from 7 AM till 8 PM throughout the experiment to ensure a minimum light intensity of 250 μmol/s/m^2^ at the canopy level. In the 4^th^ week (counting from the seed imbibition), the seedlings were transplanted into soil (local luvisol taken at Meckenheim, Germany; 50.6°N, 7.0°E; pH 7.39, density: 1.3 kg/L, N content: 0.49‰, C content: 4.38‰, K: 3.91 mg/100 g, P: 0.83 mg/100 g, Mg: 8.96 mg/100 g) filled in a plastic pot (approx. 5 x 5 x 7 cm; length, width, height). In the 6^th^ week, two plants of the same genotype were transplanted into a pot containing 5 L of the above-mentioned soil. All the irrigation was conducted using rainwater stored in an outside reservoir container to simulate natural conditions. Rainwater was sprayed on the phyllosphere 3–4 times a week until the onset of ozone fumigation to support the formation of the microbial community. Each plant received 5 g of compound fertilizer Ferty 3 (15% N, 10% P_2_O_5_, 15% K_2_O; Planta Düngemittel GmbH, Regenstauf, Germany) in three splits before the onset of the fumigation. In the 12^th^ week (*i*.*e*. in the middle of ozone fumigation), 1 g of urea was added to each pot to supply additional nitrogen. The average temperature was 29°C /25°C (day/night) and the average relative air humidity was 39% /49% (day/night). The temperature was adjusted by occasionally opening the roof of the greenhouse, which enabled the airborne microbes to enter the greenhouse.

### Stress treatment

The ozone treatment was conducted from the 10^th^ week upon seeding using custom-made ozone generators and open-top chambers as described earlier [37]. The size of the chambers was 1 x 1 x 1.3 m (length, width, height) and the sides were covered with transparent plastic sheets. Four chambers were randomly assigned to ozone treatment, and another four were used as control. Each chamber contained one pot of NB and L81, respectively. The treatment was conducted for 7 h (9 AM– 4 PM) every day for 30 d. The ozone concentration measured by an independent ozone monitor (Series 500, Aeroqual Ltd., Auckland, New Zealand) was 85 ± 34 ppb (average of 9 AM– 4 PM) and 5 ± 4 ppb in the control. The most recently fully expanded leaf of one plant in each pot was sampled from the main culm 1 d prior to the end of ozone treatment, flash-frozen with liquid nitrogen and used for expression analysis of an ozone inducible gene to confirm the effect of ozone.

### RNA extraction and gene expression analysis of plant leaf samples

The frozen leaf tissue was ground into a fine powder with mortar and pestle. Total RNA extraction and reverse transcription were conducted using PeqGOLD Plant RNA Kit (Peqlab, Erlangen, Germany) and GoScript Transcription System (Promega, Mannheim, Germany) as described earlier [37]. Real-time PCR was conducted using the StepOnePlus real-time PCR system (Applied Biosystems, Foster City, CA, USA) using the delta-delta C_T_ quantification method. The primers for the internal control (*U2_snRNP*; [38]) were 5’-CACAACAGGCCAACTGTGTC-3’ (forward) and 5’-GAGGGTCTCAACCTCACCAA-3’ (reverse), and the primer pairs for the target gene *OsNPR1* were 5’-AGAAGGGACCCACAACTCGG-3’ (forward) and 5’-TCCTCGCCAAAGCAACTCGG-3’ (reverse). The amplification efficiency of each primer pair was more than 80%.

### Microbe harvesting

At the end of the ozone treatment, microbes were harvested from the phyllosphere and rhizoplane separately from each plant. We focused on epiphytes and not endophytes by adopting a previously published “surface washing strategy” [3] rather than grinding the whole tissue. Phyllosphere bacteria were harvested as follows: Plants were cut at 20–25 cm height from the soil surface and put into a plastic bag with 250 mL of TE buffer containing detergent (10 mM Tris-HCl and 1 mM EDTA, pH 7.5 + 0.2% Silwet L-77) to efficiently collect microbes from the hydrophobic surface of rice leaves. The plastic bag containing the buffer and plant materials was shaken for 5 s, followed by two cycles of 45 s sonication using a sonication bath (35 kHz, Sonorex Super RK102H, BANDELIN electronic, Berlin, Germany) and 30 s of shaking. The resultant buffer containing washed microbes was filtrated over a mesh (mesh size 200 μm; Meerwassershop, Taufkirchen, Germany) to remove large tissue debris. The washed solution from two individual plants of the same pot was pooled, dispensed into 50-mL plastic centrifuge tubes, and centrifuged at 3,250 g, 4°C for 10 min. The pellet was resuspended in 1 mL of TE buffer (without detergent), transferred to a 2-mL centrifuge tube, and the pellet was collected again after centrifugation at 15,000 g, 4°C for 5 min. The obtained pellet was stored at -20°C until DNA extraction.

The rhizoplane microbes were collected as follows: After roughly removing the bulk soil, roots were washed thoroughly with running water to remove all adhering soil particles. After washing, a representative part of the root was excised (approx. 1/4 of the whole root) and put into a glass jar with approximately 30 g of glass beads. After addition of 200 mL TE buffer containing 0.1% Silwet L-77, roots were shaken at 300 rpm for 20 min on a KS-15 shaker (Edmund Bühler GmbH, Hechingen, Germany), followed by 5 min sonication in a sonication bath (45 kHz, USC-T 500, VWR, Darmstadt, Germany). The resulting suspension was filtered over a 500 μm nylon mesh (Meerwassershop) and centrifuged at 3,200 g, 4°C for 20 min. Washing and sonication of the roots was repeated once and the resulting pellet was merged with the pellet from the first cycle. The combined pellets were resuspended in TE buffer and stored at -20°C until DNA extraction.

### DNA extraction and amplification of 16S rRNA gene

DNA from phyllosphere microbes was extracted using a kit (FastDNA SPIN Kit; MP Biomedicals, Santa Ana, CA, USA) according to the manufacturer’s instructions with minor modifications during cell lysis and purification steps as published previously [39]. The extracted DNA was defined as “phyllosphere DNA”. The DNA from the rhizoplane microbes was extracted using the PowerSoil DNA Isolation Kit (Mo Bio Laboratories, Carlsbad, CA, USA) using the same mechanical cell lysis procedure as above. The extracted DNA was defined as “rhizoplane DNA”.

The 16S rRNA gene was amplified by PCR using primers 799F and 1193R [40]. This primer pair amplifies the V5, V6 and V7 hypervariable regions of the bacterial 16S rRNA gene, with minimum contamination of plastid DNA [40,41]. Although the selected primer pair amplifies mitochondrial DNA as well, it can easily be separated by different amplicon sizes on agarose gels [40]. For the amplification step, we used proof-read polymerase, which introduces minimal sequence errors [42]. The following setup was used for PCR reaction. For phyllosphere DNA: 2.5 μL 10x AccuPrime *Pfx* reaction mix, 0.5 μL AccuPrime *Pfx* DNA polymerase (Thermo Fisher Scientific, Waltham, MA, USA), 0.5 μL of 10 μM 1193R primer, 0.5 μL of 10 μM 799F primer (attached with sample specific barcode sequences; S1 Table), 19.3 μL of nuclease-free water and 2 μL of phyllosphere DNA template. The thermal condition for PCR was the following: 95°C for 2 min, 35 cycles of 95°C for 15 s, 50°C for 30 s and 68°C for 1 min, followed by an additional extension at 68°C for 5 min. For rhizoplane DNA, the amount of template DNA was reduced to 1 μL since using a large amount of template from the rhizoplane fraction adversely affected the amplification, probably due to a higher content of PCR inhibitory substances in this fraction. The PCR was performed with 20 cycles.

The amplified DNA fragments were separated on an agarose gel. The band representing the bacterial 16S rRNA gene fragment was excised and purified using a gel-extraction kit (FastGene Gel/PCR Extraction Kit; Nippon Genetics, Dueren, Germany). The DNA concentration of the elution was measured using a Qubit Fluorometer (Thermo Fisher Scientific). Equimolar amounts of each elution were separately pooled for phyllosphere and rhizoplane. The pools were purified with magnetic beads following manufacturer’s instructions (HighPrep PCR beads, MagBio Genomics, Gaithersburg, MD, USA). Library preparation using the NEBNext Ultra^™^ DNA Library Prep Kit (New England Biolabs, Ipswich, MA, USA) and sequencing were performed by the Max Planck-Genome-centre Cologne. Sequencing was conducted on an Illumina MiSeq personal sequencer using the v2 chemistry with paired-end reads of 2 x 250 bp following the manufacturer’s instructions (Illumina, San Diego, CA, USA). The MiSeq sequence data were deposited in the NCBI SRA archive (accession number SRP075957).

### Data processing and bioinformatics analyses

Since the Illumina MiSeq sequencing run generated paired-end reads, the full-length amplicon was reconstructed by combining two reads by PANDAseq software [43], applying the quality threshold of 0.9. Most of the downstream processing was conducted using the Mothur v1.36.1 programme [44], largely following the Standard Operating Procedure for MiSeq (www.mothur.org/wiki/MiSeq_SOP; [45]). Demultiplexing and quality control were conducted, allowing perfect match of the barcode sequences, average quality score > 33, no ambiguous base, not more than 8 bp of homopolymer, and a length between 370 and 380 bp (after removal of primers and barcode sequences), since most of the expected amplicons ranged from 370 to 380 bp length (judged by TaxMan database; http://www.ibi.vu.nl/programs/taxmanwww/; [46]). Chimeric sequences were removed using UCHIME v4.2 [47]. Taxonomy was assigned to each read by using the RDP_trainset v14 database [48], and the reads not classified as Bacteria were removed. The reads were aligned using the Silva bacteria sequence database v119 [49]. After removing singletons, the reads were clustered into operational taxonomic units (OTUs) at 97% identity using the cluster.split command, applying taxlevel = 4 and cutoff = 0.15. Rarefaction curves were created with the rarefaction.single command with 1,000 iterations. Alpha diversity indices were calculated using the summary.single command after subsampling, adjusting to the minimum sample read number (54,238 for phyllosphere and 62,188 for rhizoplane). Analysis of molecular variance (AMOVA) and homogeneity of molecular variance (HOMOVA) were calculated by the implementation of Mothur based on the Yue and Clayton measure of dissimilarity distance matrix among samples [50]. Weighted UniFrac distance [51] was calculated using the phylogenetic tree among OTUs and a count file containing the information of the abundance of each OTU in each sample. Analysis of similarity (ANOSIM) [52] was conducted after the clustering using the implementation of Mothur.

Community functional analysis was conducted using the PICRUSt programme [53] on the Galaxy platform [54]. Since PICRUSt only allows the use of the Greengenes reference taxonomy database, the above analysis was carried out again using Greengenes 13 database [55] instead of RDP reference taxonomy files. The KEGG database [56,57] was used to map the genes on the metabolic pathway.

### Statistical analyses

Statistical analyses of diversity indices were conducted using SAS software (SAS Institute, Cary, NC, USA) applying a PROC MIXED model. Treatment, genotype and the interaction between the two of them were treated as fixed effects, and chamber was treated as random effect. For relative abundance of OTU and PICRUSt analyses, an R-based programme edgeR [58] was used to determine the effect of treatment and genotype since these analyses produced discrete data, not continuous data. For multiple comparisons, a false discovery rate (FDR) was calculated according to Benjamini and Hochberg [59].

## Results

### Ozone treatment and next-generation sequencing

**Fig 1:**
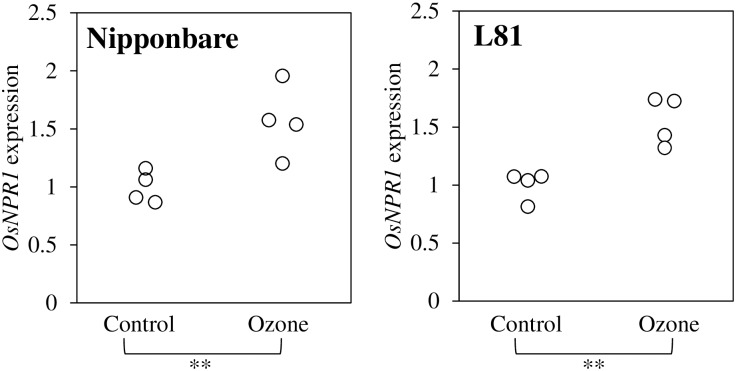
Relative expression level of an ozone marker gene *OsNPR1*. The expression level of the *OsNPR1* gene (*Os01g0194300*) was analysed from each sample to assess the effect of ozone fumigation on the whole plant. Total RNA was extracted from the most recently fully expanded leaf from one plant in each pot and qPCR was conducted. The values for 16 samples (four different groups and four replicates) are shown. The expression level was calibrated to an internal control gene *U2_snRNP* (*Os05g0564200*). The mean value obtained for *OsNPR1* from the four control samples was set to 1 in each genotype. The asterisks indicate that the expression levels are significantly different between control and ozone treated samples (*P* < 0.01; by Student’s *T*-test).

Rice plants were treated with 85 ppb of ozone stress for 30 d prior to the microbe collection. This is a realistic concentration, which is anticipated in the near future in many parts of the world, and this high level has already been reached in some highly polluted areas [16,20]. Ozone treatment indeed affected plants, as shown by the induced expression of *OsNPR1* (Fig 1), a general marker gene for ozone stress in rice [37]. OsNPR1 is a transcription factor which modulates a wide range of defense responses in rice [60], and which is stimulated under ozone stress. The Illumina MiSeq sequencing yielded a total of 7,935,598 reads for pooled phyllosphere samples and 7,747,296 reads for rhizoplane samples. Through stringent read quality check and removal of chimeric and singleton reads, we obtained 2,256,695 high-quality reads for the 16 phyllosphere samples and 1,944,731 high-quality reads for the 16 rhizoplane samples (S2 Table). These reads were used for defining operational taxonomy units (OTUs) at 97% identity and further analyses. One of the 16 samples showed an excessively high number of OTUs in the phyllosphere fraction. Moreover, this sample was most distantly related to all other samples when Yue and Clayton dissimilarity distance [50] was calculated both in the phyllosphere and rhizoplane (S1 Fig). Therefore, we treated this sample as an outlier and eliminated this sample from both, the phyllosphere and rhizosphere dataset, for all subsequent analyses.

### Community structure and diversity analysis

**Fig 2:**
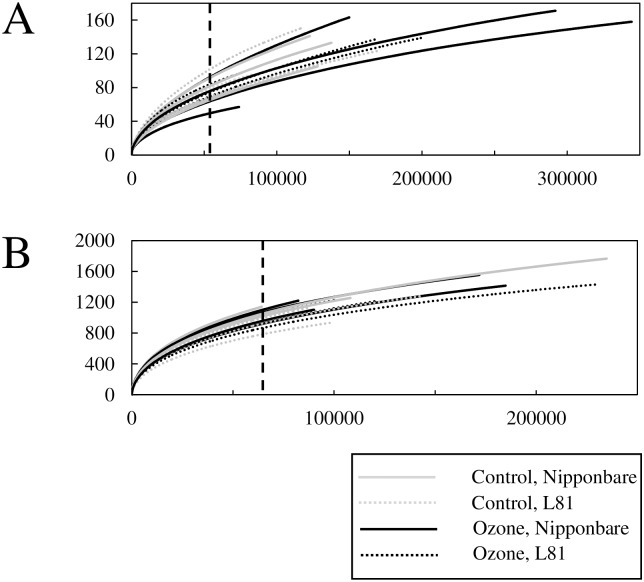
Rarefaction curve of each sample. The number of sampled sequences is plotted on the x-axis, and the number of different OTUs detected in the samples is plotted on the y-axis. (A) phyllosphere, (B) rhizoplane. The dashed vertical line indicates the depth of subsampling (54,238 for phyllosphere and 62,188 for rhizoplane). The number of replicates was three for ozone-L81 group and four for the rest of the groups.

Based on OTUs picked at 97% identity, alpha-diversity in each group was visualized by rarefaction curves (Fig 2). In both compartments, sequencing effort was reasonably deep to thoroughly analyse the community (Fig 2). Comparing the two compartments, the rhizoplane was inhabited by a higher diversity of bacteria. No clear separation was observed among the groups in either fraction, meaning that different genotype or treatment did not cause obvious changes in the diversity and richness of the microbial community. Alpha diversity was also quantitatively assessed by calculating inverse Simpson index. It was also not significantly affected by different genotypes or ozone treatment (Table 1). Species richness and evenness were further analysed by calculating the Chao 1 index and Simpson’s evenness index, respectively. Ozone treatment or different genotypes did not significantly affect these indices (Table 1). Other indices showing the community’s diversity and evenness also did not reveal any significant effect of treatment or genotypes (S3 Table).

**Table 1:** Comparison of various alpha diversity indices in different groups and the result of ANOVA in the phyllosphere and the rhizoplane.

Fraction	Category	Index	NB-Control	L81-Control	NB-Ozone	L81-Ozone	G	T	GxT
**Phyllosphere**	**Diversity**	**Inverse of Simpson index**	1.22 ± 0.04	1.22 ± 0.05	1.42 ± 0.17	1.29 ± 0.07	ns	ns	ns
	**Richness**	**Chao1 index**	244 ± 35	179 ± 25	234 ± 46	254 ± 50	ns	ns	ns
	**Evenness**	**Simpson’s evenness index**	0.011 ± 0.001	0.012 ± 0.002	0.012 ± 0.003	0.011 ± 0.001	ns	ns	ns
**Rhizoplane**	**Diversity**	**Inverse of Simpson index**	9.7 ± 1.6	8.7 ± 2.5	7.0 ± 1.4	7.7 ± 1.6	ns	ns	ns
	**Richness**	**Chao1 index**	1948 ± 224	1717 ± 113	2025 ± 130	2000 ± 65	ns	ns	ns
	**Evenness**	**Simpson’s evenness index**	0.0087 ± 0.0023	0.0080 ± 0.0023	0.0055 ± 0.0012	0.0061 ± 0.0014	ns	ns	ns

Alpha diversity indices were categorized into “Diversity”, “Richness” and “Evenness”, and the values of each index were calculated for four groups (*i*.*e*. two treatments and two genotypes). ANOVA was conducted specifying treatment, genotype and the interaction between them as variables. The mean value of three or four replicates is shown with standard error. The result of ANOVA is shown on the right columns. G, genotype; T, treatment; GxT, genotype and treatment interaction; n.s., not significant. NB, Nipponbare.

In order to further evaluate a potential effect of ozone treatment and genotypic differences, we analysed the structure of the community by analysis of molecular variance (AMOVA) and homogeneity of molecular variance (HOMOVA) [61]. AMOVA, a nonparametric analogue of ANOVA, which determines whether the genetic diversity within each community is different from the average genetic diversity of both communities, revealed no significant effect of genotype or treatment (Table 2). In contrast, HOMOVA, a nonparametric analogue of Bartlett’s *F*-test, which is used to test whether the genetic diversity is the same in multiple communities, showed a significant treatment effect in the phyllosphere fraction (Table 2). The variance within the group was higher in ozone-treated samples (1.7 x 10^−4^) compared to that of the control samples (9.1 x 10^−6^).

**Table 2:** Comparison of community structure by analysis of molecular variance (AMOVA) and homogeneity of molecular variance (HOMOVA) and the results of significance tests.

Fraction	Parameter	Comparison	*P* value
**Phyllosphere**	**AMOVA**	Control vs Ozone	0.675
		Nipponbare vs L81	0.348
	**HOMOVA**	Control vs Ozone	0.021
		Nipponbare vs L81	0.330
**Rhizoplane**	**AMOVA**	Control vs Ozone	0.161
		Nipponbare vs L81	0.313
	**HOMOVA**	Control vs Ozone	0.146
		Nipponbare vs L81	0.412

AMOVA and HOMOVA were conducted by the Mothur programme based on the distance matrix created by the Yue and Clayton measure of dissimilarity made from the subsampled OTU table. The comparison was performed between different genotypes and ozone treatment. Analysis was conducted separately for each fraction.

**Fig 3:**
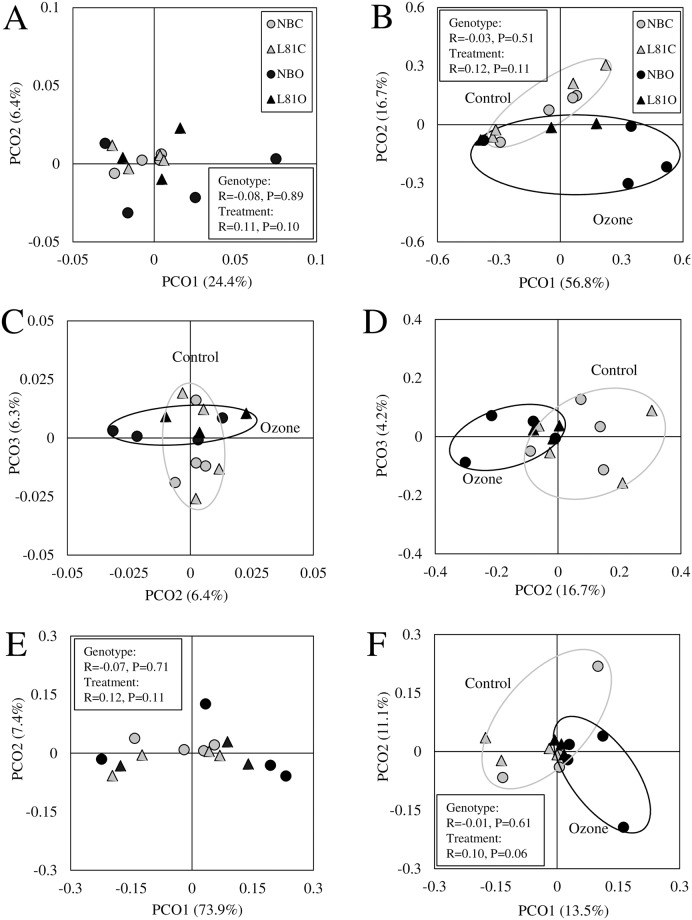
Principal coordinate analysis plot of Yue and Clayton and weighted UniFrac distances among samples. (A-D) Relatedness among samples shown by the principal coordinate analysis plot based on the Yue and Clayton dissimilarity distance in the phyllosphere fraction (A, C) and rhizoplane fraction (B, D). The first and second axes were plotted for A and B, and the second and third axes were plotted for C and D. (E, F) Principal coordinate analysis plot of weighted UniFrac analysis in the phyllosphere fraction (E) and rhizoplane fraction (F) based on the phylogenetic tree of OTUs and their abundance in each sample. The results of analysis of similarity (*R* value and *P* value) are also shown. An ellipse was drawn when a tendency of clustering between the treatments was seen on the plot. NB, Nipponbare; C, control; O, ozone. The number of replicates was three for L81O group and four for the rest of the groups.

The relationship among samples was visualized in a principal coordinate analysis (PCoA) plot based on the Yue and Clayton measure of dissimilarity [50] (Fig 3A–3D). Plotting the first two axes, a weak clustering was observed for the rhizoplane fraction (Fig 3B). In the phyllosphere fraction, a somewhat weak clustering was observed by plotting the second and the third axes (Fig 3C). The significance of clustering was analysed by calculating the values for analysis of similarity (ANOSIM). In both fractions, no genotypic effect (*R* = -0.08, *P* = 0.89 for the phyllosphere, *R* = -0.03, *P* = 0.51 for the rhizoplane) was observed, while a weak treatment effect (*R* = 0.11, *P* = 0.10 for the phyllosphere, R = 0.12, P = 0.11 for the rhizoplane) was observed for both fractions (Fig 3A–3D). A dendrogram made from the same distance matrix showed a similar pattern (S2 Fig).

To obtain deeper insight into the changes in the community composition, we calculated a weighted UniFrac distance. UniFrac makes use of the phylogenetic information of the detected OTUs and provides more power than simply comparing the abundance of OTUs. The weighted UniFrac analysis was conducted on the subsampled OTU table for each fraction (the subsampling size was 54,238 for phyllosphere and 62,188 for rhizoplane, including all rare OTUs). The PCoA plot based on the UniFrac distance matrix showed no obvious clustering in the phyllosphere fraction, although the associated *P* value was relatively small (*P* = 0.12; Fig 3E). In the rhizoplane fraction, there was a weak tendency of clustering (*P* = 0.06, *R* = 0.10 by ANOSIM).

### Community compositional analysis

**Fig 4:**
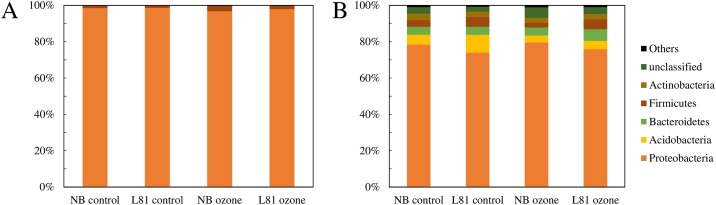
Relative abundance of each phylum in different environments. The phyla with more than 0.5% of relative abundance are shown for each group. (A) phyllosphere, (B) rhizoplane. NB, Nipponbare. The value is the mean of three to four replicates.

To evaluate possible responses of the microbial community members further, the relative abundance of each OTU was calculated for each fraction. Here we defined OTUs with more than 0.5% of relative abundance as abundant OTUs (A-OTU), and the subsequent community compositional analysis was conducted on A-OTUs, since rare OTUs cannot be robustly quantified due to low replicability [62]. The phyllosphere fraction contained 6 A-OTUs, while the rhizoplane fraction contained 23 A-OTUs (S4 and S5 Tables). The phyllosphere community was largely dominated by one OTU classified as *Variovorax* at genus level, comprising 89% of the total reads (S4 Table). On the other hand, the major OTUs in the rhizoplane were classified as *Azospira* (28%), *Pelomonas* (13%), *Dechloromonas* (7%) and *Geothrix* (5%) at genus level, and these four major OTUs accounted for 50% of the total reads (S5 Table). We calculated the effect of different genotypes and treatment on the relative abundance of each A-OTU. In the phyllosphere fraction, no A-OTU was significantly affected when false-discovery rate (FDR) adjustment was applied (S4 Table). In the rhizoplane fraction, on the other hand, two OTUs were significantly affected by ozone treatment (S5 Table). An OTU belonging to the *Rhodospirillaceae* family and another OTU belonging to the *Clostridiales* order decreased their relative abundance in ozone-treated plants as compared with the plants under the control condition (FDR < 0.05; S5 Table). We further compared the relative abundance on higher levels of taxonomic classification (*i*.*e*. family, order, class and phylum; Fig 4; S4 and S5 Tables). No taxa were significantly affected by treatment or different genotypes when the OTUs were classified by these higher taxonomy levels (S4 and S5 Tables).

### Community functional analysis

To explore the possibility that the function of the community alters due to these effects, we estimated the number of genes involved in certain functions or metabolic pathways, which each community possesses as a whole. To this end, we used the PICRUSt software [53], which estimates the functional profile of the community based on the abundance of genes involved in certain metabolic pathways in each taxon and their relative abundance. We selected genes with counts of more than 1,000 in all the samples for robust quantification and conducted statistical analysis using the edgeR programme. None out of 1,897 and 2,750 pathways for the phyllosphere and rhizoplane fractions, respectively, were significantly affected by ozone treatment or different genotypes (S6 and S7 Tables).

## Discussion

### Evaluation of the sequencing analysis and dominant taxa in each fraction

Our results of the relative abundance of the rhizoplane community members are largely comparable with a previous report conducted under field and greenhouse conditions, where *Proteobacteria* were the dominant phylum, and *Acidobacteria* and *Actinobacteria* were also relatively abundant in the rice rhizoplane [63] (Fig 4; S5 Table). This suggests that a characteristic rhizoplane microbiota established under the conditions adopted in the current study. *Chloroflexi* were also reported as an abundant phylum earlier [63], but not in the current study (S5 Table). This inconsistency and some other differences (such as a rather low abundance of *Plantomycetes*) might originate from different soil types, growth conditions or different primers used in different studies. In previous studies, it was shown that different primers caused biases in the relative abundance of some taxa [62,64]. In the phyllosphere fraction, the community was dominated by the genus *Variovorax*, a member of the class *Betaproteobacteria* (S4 Table). Previous studies identified *Actinobacteria* and *Alphaproteobacteria* [3] or *Enterobacteriaceae* (classified as *Gammaproteobaceria* at the class level) [65] as the dominant taxa in the phyllosphere in paddy-grown rice. Such discrepancies can be explained by the fact that the phyllosphere bacterial community is highly influenced by the initial community members, leading to the formation of specific microbial communities (”stochastic niche model”) [66]. Considering that the phyllosphere bacteria originate from diverse sources including air, soil, paddy water and the seed itself [3,4,66], this fraction is probably more sensitive to the environmental factors and plant growth conditions, which may affect the initial colonizers. Since some of the species in the *Variovorax* family inhabit soil and paddy water [67,68], the large number of *Variovorax* in the phyllosphere could have originated from the irrigation or paddy water. However, the high abundance of *Variovorax* is not simply a passive effect of irrigation, since bacteria of this taxon actively form habitats as the plant grows and increase their relative abundance [66]. Another reason for the discrepancy could be different growth conditions and the geographical locations, since our experiment was conducted in a greenhouse at a location different from the natural rice cultivation areas.

### Ozone treatment leads to weak responses in the bacterial community composition

HOMOVA revealed a significant effect of ozone on the phyllosphere microbial community (Table 2), suggesting that the microbial communities from the phyllosphere of ozone-treated plants are more variable than those from the phyllosphere of control plants. The difference of the community composition was also supported by the PCoA plot based on the Yue and Clayton dissimilarity index, where the two treatments were weakly clustered in both fractions (Fig 3A–3D). In weighted UniFrac analysis, very weak treatment effects were observed in the rhizoplane fraction (Fig 3E and 3F). We found that two taxa were significantly affected by ozone treatment in the rhizoplane. *Rhodospirillaceae* are nonsulfur photosynthetic bacteria, and often found in freshwater-rich conditions such as mud. The order *Clostridiales* belongs to the phylum *Firmicutes*, and it is an obligate anaerobe often found in flooded soil. The relationship between these bacterial taxa and the response of plants to ozone needs further investigation.

Despite the observed changes in relative abundance of the above-mentioned taxa, no significant treatment effect was seen in the molecular functions of the community (S6 and S7 Tables). Considering these facts, we suggest that ozone affects the community as a whole, but not towards a certain direction, which would lead to an alteration of the function of the formed community in either fraction.

The fact that ozone treatment affected rhizoplane community composition at all is remarkable, as it is less intuitive than effects observed in the phyllosphere. The overall effect of ozone was more evident in the rhizoplane fraction than in the phyllosphere fraction (Fig 3). It is unlikely that effects in the rhizosphere were direct, since ozone is mostly taken up through stomata and immediately decomposes into reactive oxygen species (ROS). Therefore the concentration of ozone is virtually zero beyond the stomatal pore [69], and ozone does also not penetrate into the soil [28]. Earlier investigations revealed that elevated tropospheric ozone leads to alterations of mycorrhizal colonization in the rhizosphere (reviewed in [70]). Recently, field experiments demonstrated changes in the methanogenic archaeal and bacterial community in the rhizosphere soil of rice under elevated ozone concentration. It was found that an elevated ozone concentration affects the relative abundance of some microbial taxa and decreases phylogenetic diversity in the rhizosphere of rice paddy soil [25–27]. A previous study suggested changes in carbon sequestration in the soil under elevated ozone concentration as an indirect effect of ozone through changes in microbial community composition [71]. Thus, our current results are consistent with previous findings, where ozone significantly affected belowground microorganisms [25,71–74]. However, the decrease of bacterial diversity in the rhizosphere reported previously [26] was not seen in the current study in the rhizoplane. This might be due to different ozone treatment schemes or different growth stages of the plants adopted in different studies, since the response of the rhizosphere microbial community was stronger in an earlier growth stage [26,27]. Another explanation is linked to the fact that different colonizers inhabit rhizosphere soil and rhizoplane; While the rhizosphere bacterial community has close links to the bulk soil bacteria, the rhizoplane bacteria form a unique community, which is different from that of the rhizosphere in terms of composition and complexity [63].

### Possible factors leading to different community composition in the phyllosphere

A possible mechanism that has the potential to induce changes in phyllosphere bacterial communities is via carbon release from the leaf surface. Plants emit volatile organic compounds upon ozone stress to scavenge incoming ozone before passing through the stomata [75]. Since some phyllosphere bacteria utilize volatile organic compounds emitted from plants [4], different carbon availability on the leaf surface under ozone stress might have affected the nutrient availability of the phyllosphere microorganisms. However, this may not explain the type of response observed in the current study, since bacteria that utilize VOCs are rather specific taxa [4,76,77], and it would have affected the relative abundance of these VOC-utilizing taxa and their VOC-related metabolic pathways between treatments. Another possibility for community compositional changes is direct toxicity of ozone. Previous studies reported that bacteria and fungi were killed by direct exposure to ozone in the air [29,30]. However, these strong negative effects of ozone on microbes were observed at rather high concentrations of ozone, and the involvement of a direct effect remains unclear at lower concentrations as applied in the current study. Possible mechanisms should be investigated in more detail in the future.

### Possible factors leading to different community composition in rhizoplane/rhizosphere

Since the rhizosphere/rhizoplane microbes utilize the root exudates and nutrients from the root surface, different allocation of carbon and nutrient status of rhizosphere/rhizoplane upon ozone stress might have affected the community composition [78]. Considering that a significant amount of net photosynthetically assimilated carbon is partitioned into the rhizosphere as root exudates and tissue sloughing (up to 50% of assimilated carbon; [5,79]) and that ozone negatively affects carbon assimilation [24,80], altered nutrient availability from the plant root might explain the effect of ozone on the rhizosphere/rhizoplane microbial community. Changes in root morphology represent another possibility. Several reports suggested that ozone decreases the amount of fine roots in some plant species [81,82]. Moreover, a previous study reported different levels of fungal colonization on different root types in rice [83,84]. Therefore, different sets of microorganisms might have colonized different root types (*e*.*g*. fine roots and crown roots), which were differently affected by ozone treatment.

### Limitation of the current study and future perspectives

The current approach did not take into account archaea and fungi since the primer pair that we used did not amplify their 16S rRNA genes. Although the abundance of these groups of organisms is smaller compared to that of bacteria in the phyllosphere and rhizosphere/rhizoplane [1–4,31,32], they might be involved in additional important processes such as nutrient uptake (*e*.*g*. arbuscular mycorrhizal fungi) and methane emission (*e*.*g*. methanogenic archaea). A further limitation of the current approach is that the analysis was largely limited to epiphytes, and endophytes were not systematically collected during the sampling. Since ozone induces oxidative stress and leads to the formation of ROS in the apoplast [23,85] and bacteria are liable to damage under such conditions [86,87], some endophytes colonizing the apoplast may be also affected by ozone stress. Also, ozone stress increases the expression and protein levels of some anti-pathogenic enzymes such as β-glucanases and chitinases in various plant species [88–90], which could also affect the endophytic microbial community. Finally, it has to be considered that the experimental conditions in the current study were different from natural rice cultivating areas, due to different soil types, use of a greenhouse and the geographical location of the experimental site.

In conclusion, we detected a weak effect of ozone on the bacterial community in the phyllosphere and the rhizosphere of rice as judged by HOMOVA and clustering analyses, although this difference did not lead to detectable changes in specific metabolic pathways. Further research is warranted to elucidate the underlying mechanisms for the shift in community changes.

## Supporting Information

S1 Fig Principal coordinate analysis plot based on the Yue and Clayton distance among the samples in the phyllosphere and rhizoplane fraction. (DOCX)

S2 Fig Relatedness among samples calculated by the Yue and Clayton dissimilarity distance in the phyllosphere and the rhizoplane. (DOCX)

S1 Table Primers used for the amplification of the 16S rRNA gene. (DOCX)

S2 Table Thirty-two DNA samples (*i*.*e*. phyllosphere and rhizoplane DNA from 16 different pots) and their sequence read number in each fraction. (DOCX)

S3 Table Comparison of alpha diversity indices in different groups and the result of ANOVA in the phyllosphere and the rhizoplane. (DOCX)

S4 Table Relative abundance of each taxon (>0.5%) in the phyllosphere fraction at different classification levels in different treatments and genotypes. (XLSX)

S5 Table Relative abundance of each taxon (>0.5%) in the rhizoplane fraction at different classification levels in different treatments and genotypes. (XLSX)

S6 Table Estimated number of genes involved in different pathways in different groups of samples in the phyllosphere. (XLSX)

S7 Table Estimated number of genes involved in different pathways in different groups of samples in the rhizoplane. (XLSX)
