# Supplementary material for: Effects of Elevated Tropospheric Ozone Concentration on the Bacterial Community in the Phyllosphere and Rhizoplane of Rice
Source: PLoS One. 2016 Sep 19;11(9):e0163178. doi: 10.1371/journal.pone.0163178 (PMC5028031; doi:10.1371/journal.pone.0163178)
Supplement: S1 Fig — (DOCX) [file pone.0163178.s001.docx]

**S1 Fig. Principal coordinate analysis plot based on the Yue and Clayton distance among the samples in the phyllosphere and rhizoplane fraction.**

Principal coordinate plots were created based on the Yue and Clayton distance matrix. (A) phyllosphere, (B) rhizoplane. The first two axes are plotted. NB, Nipponbare; C, control; O, ozone. The data point surrounded by a dashed grey circle was not included in the subsequent analyses, since this sample is most distantly related to all other samples (see main text).
