# Supplementary material for: Effects of Elevated Tropospheric Ozone Concentration on the Bacterial Community in the Phyllosphere and Rhizoplane of Rice
Source: PLoS One. 2016 Sep 19;11(9):e0163178. doi: 10.1371/journal.pone.0163178 (PMC5028031; doi:10.1371/journal.pone.0163178)
Supplement: S2 Fig — (DOCX) [file pone.0163178.s002.docx]

**S2 Fig. Relatedness among samples calculated by the Yue and Clayton dissimilarity distance in the phyllosphere (A) and the rhizoplane (B).**

The dendrogram was constructed by the Mothur programme based on the distance matrix among samples after removing one outlier sample. Each symbol represents samples from different environments. The branch length indicates the distance as defined by 1-θ (theta), where θ (theta) shows the similarity index as calculated by the method of Yue and Clayton. NB, Nipponbare.
