# Supplementary material for: Effects of Elevated Tropospheric Ozone Concentration on the Bacterial Community in the Phyllosphere and Rhizoplane of Rice
Source: PLoS One. 2016 Sep 19;11(9):e0163178. doi: 10.1371/journal.pone.0163178 (PMC5028031; doi:10.1371/journal.pone.0163178)
Supplement: S1 Table — (DOCX) [file pone.0163178.s003.docx]

**S1 Table. Primers used for the amplification of the 16S rRNA gene.**

A specific barcode sequence (underlined) was connected to primer 799F in each sample. M, A or C; K, G or T. Each primer pair was used for the phyllosphere and corresponding rhizoplane DNA from the same pot.
