# Supplementary material for: Effects of Elevated Tropospheric Ozone Concentration on the Bacterial Community in the Phyllosphere and Rhizoplane of Rice
Source: PLoS One. 2016 Sep 19;11(9):e0163178. doi: 10.1371/journal.pone.0163178 (PMC5028031; doi:10.1371/journal.pone.0163178)
Supplement: S2 Table — (DOCX) [file pone.0163178.s004.docx]

**S2 Table. Thirty-two DNA samples (*i.e.* phyllosphere and rhizoplane DNA from 16 different pots) and their sequence read number in each fraction.**

| Genotype | Treatment | Replicate | #HQ read (Phyllosphere) | #HQ read (Rhizoplane) |
| --- | --- | --- | --- | --- |
| Nipponbare | Control | 1 | 137,639 | 234,820 |
| Nipponbare | Control | 2 | 128,164 | 108,114 |
| Nipponbare | Control | 3 | 122,794 | 62,188 |
| Nipponbare | Control | 4 | 84,669 | 64,160 |
| Nipponbare | Ozone | 1 | 344,353 | 171,891 |
| Nipponbare | Ozone | 2 | 73,920 | 184,875 |
| Nipponbare | Ozone | 3 | 291,891 | 90,062 |
| Nipponbare | Ozone | 4 | 149,829 | 82,315 |
| L81 | Control | 1 | 168,978 | 143,204 |
| L81 | Control | 2 | 54,732 | 99,178 |
| L81 | Control | 3 | 84,294 | 86,957 |
| L81 | Control | 4 | 118,046 | 70,959 |
| L81 | Ozone | 1 | 75,124 | 122,015 |
| L81 | Ozone | 2 | 200,379 | 231,218 |
| L81 | Ozone | 3 | 167,645 | 101,136 |
| L81* | Ozone | 4 | 54,238 | 91,639 |
|  |  | Total | 2,256,695 | 1,944,731 |

* This sample was omitted from the analysis. See the Results section for detail.

The number of high-quality (HQ) reads from both fractions is shown with corresponding genotype, treatment and replicate number. The bottom row indicates the total read number, which is the sum of the read numbers from all samples.
