# Supplementary material for: Effects of Elevated Tropospheric Ozone Concentration on the Bacterial Community in the Phyllosphere and Rhizoplane of Rice
Source: PLoS One. 2016 Sep 19;11(9):e0163178. doi: 10.1371/journal.pone.0163178 (PMC5028031; doi:10.1371/journal.pone.0163178)
Supplement: S3 Table — (DOCX) [file pone.0163178.s005.docx]

**S3 Table. Comparison of alpha diversity indices in different groups and the result of ANOVA in the phyllosphere and the rhizoplane.**

| **Fraction** | **Category** | **Index** | **NB- Control** | **L81- Control** | **NB- Ozone** | **L81- Ozone** | **G** | **T** | **GxT** |
| --- | --- | --- | --- | --- | --- | --- | --- | --- | --- |
| **Phyllosphere** | **Diversity** | **Shannon index** | 0.50 ± 0.06 | 0.48 ± 0.08 | 0.68 ± 0.19 | 0.58 ± 0.10 | ns | ns | ns |
|  | **Evenness** | **Shannon’s evenness index** | 0.10 ± 0.01 | 0.10 ± 0.01 | 0.14 ± 0.04 | 0.12 ± 0.02 | ns | ns | ns |
| **Rhizoplane** | **Diversity** | **Shannon index** | 3.7 ± 0.1 | 3.4 ± 0.2 | 3.5 ± 0.2 | 3.6 ± 0.0 | ns | ns | ns |
|  | **Evenness** | **Shannon’s evenness index** | 0.52 ± 0.00 | 0.48 ± 0.02 | 0.49 ± 0.02 | 0.50 ± 0.01 | ns | ns | ns |

The Shannon index and Shannon’s evenness index indicating the diversity and evenness of the community, respectively. ANOVA was conducted specifying treatment, genotype and the interaction between them as variables. The mean value of three or four replicates is shown with standard error. The result of ANOVA is shown on the right columns. G, genotype; T, treatment; GxT, genotype and treatment interaction; n.s., not significant. NB, Nipponbare.
